# Regularization of a half-center oscillator network by closed-loop control

**DOI:** 10.1186/1471-2202-16-S1-P275

**Published:** 2015-12-18

**Authors:** Irene Elices, Pablo Varona

**Affiliations:** 1 Grupo de Neurocomputación Biológica, Departamento de Ingeniería Informática, Escuela Politécnica Superior, Universidad Autónoma de Madrid, Madrid, 28049, Spain

## 

Central Pattern Generators (CPGs) are neural circuits that control muscle functioning by means of rhythmic patterns. These networks are usually built up on a minimal configuration based on reciprocal inhibitory connections responsible for the production of alternating spiking-bursting activity. Experimental observations in the crustacean pyloric CPG show that most neurons, when isolated, present a highly irregular, in fact chaotic, bursting activity [1-3]. This rich intrinsic dynamics provides flexibility for negotiating rhythms through the reciprocal inhibitory connections between neurons which lead to the regularization of the chaotic behavior when the neurons interact within the circuit [4].

**Figure 1:**
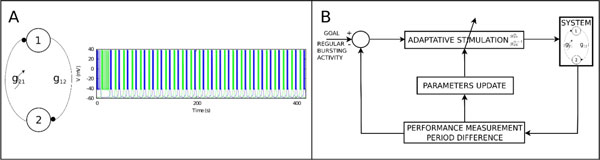
**Panel **A**: Activity of the system using the closed-loop algorithm**. Panel **B**: Schematic representation of the closed-loop protocol.

Closed-loop interactions are typically used in electrophysiological experiments using dynamic clamp protocols [5] and have been generalized for different description levels of the nervous system [6]. In this work we show that feedback protocols can also be used in theoretical studies to search for specific dynamics or explore the parameter space of a given model. We have built a CPG model based on a minimal network with two neurons connected by bidirectional fast chemical inhibitory synapses. The network generates alternating bursting activity in the neurons, which can be regular or irregular depending on the maximal conductances of the inhibitory synapses. We employ a simple adaptive closed-loop protocol to regularize the alternating chaotic activity of the model (see Figure 1 panel A). This protocol adapts online the maximal conductance of one of the synapsis to achieve the aimed regular alternating bursting activity (see Figure 1, panel B). Moreover, the closed-loop algorithm can be used to automatically map the region of maximal conductance values that lead to regular activity.
